# Risk factors for dementia onset in older adults with metastatic renal cell carcinoma

**DOI:** 10.1093/geroni/igab046.2613

**Published:** 2021-12-17

**Authors:** Samuel Miller, Lauren Wilson, Melissa Greiner, Jessica Pritchard, Tian Zhang, Deborah Kaye, Harvey Cohen, Michaela Dinan

**Affiliations:** 1 Yale University, Hamden, Connecticut, United States; 2 Duke University School of Medicine, Durham, North Carolina, United States; 3 Duke University, Durham, North Carolina, United States; 4 Duke University, Duke University, North Carolina, United States; 5 Duke Cancer Institute, Duke Cancer Institute/Durham, North Carolina, United States; 6 Yale University, New Haven, Connecticut, United States

## Abstract

Renal dysfunction is a driver of dementia. It is also associated with renal cell carcinoma, possibly the result of the tumor itself or from cancer treatment. This study evaluates metastatic renal cell carcinoma (mRCC) as a risk factor for developing mild cognitive impairment or dementia (MCI/D) as well as the impact of RCC-directed therapies on the development of MCI/D. We identified all patients diagnosed with mRCC in SEER-Medicare from 2007-2015. The main outcome was incident MCI/D within one year of mRCC diagnosis or cohort entry. Exclusion criteria included age <65 at mRCC diagnosis and diagnosis of MCI/D within preceding year of mRCC diagnosis. Patients with mRCC (n=2,533) were matched to non-cancer controls (n=7,027) on age, sex, race, comorbidities and year. Cox proportional hazards regression showed that having mRCC (HR 8.52, 95% MCI/D 6.49-11.18, p<0.001) and being older (HR 1.05 for 1-year age increase, 95% MCI/D 1.03-1.07, p<0.001) were predictive of developing MCI/D. A second Cox proportional hazards regression of only patients with mRCC revealed that neither those initiating treatment with oral anticancer agents (OAAs) nor those who underwent nephrectomy were more likely to develop MCI/D. Black patients had a higher risk of dementia compared to white patients (HR 1.92, 95% MCI/D 1.02-3.59, p=0.047). In conclusion, patients with mRCC were more likely to develop MCI/D than those without mRCC. The medical and surgical therapies evaluated were not associated with increased incidence of MCI/D. The increased incidence of MCI/D in older adults with mRCC may be the result of the pathology itself.

